# A case of DAPSONE INDUCED METHEMOGLOBINEMIA

**DOI:** 10.1186/s40545-019-0185-y

**Published:** 2019-06-20

**Authors:** Najia Mahmood, Mutti Ullah Khan, Ihtisham U. L. Haq, Farhat Afshan Jelani, Aayesha Tariq

**Affiliations:** 1 FCPS Medicine, Holy Family Hospital, Rawalpindi Medical University and Allied Hospitals, Rawalpindi, Pakistan; 2 FCPS Anesthesia, Combined Military Hospital, Sargodha, Pakistan; 3 Pakistan Atomic Energy Commission General Hospital, Islamabad, Pakistan

**Keywords:** Cyanosis, Methemoglobinemia, Oxidizing agent, Tissue hypoxia

## Abstract

Methemoglobinemia (MetHb) being a rare cause of cyanosis is generally not considered in its differential diagnosis. Methemoglobinemia is an abnormal Hb produced physiologically by auto-oxidation. If this process of auto oxidation is impaired either due to genetic defect or due to exogenous drugs/ toxins, its level starts rising. Once it is > 3%, tissue hypoxia ensues. Here is a case of dapsone induced MetHb and is reported in a young girl with central cyanosis, and was treated successfully with methylene blue. Methemoglobinemia should be considered in differential diagnoses of cyanosed patient with normal ABGs, PaO_2_ and cardio-respiratory status. If left untreated, the disease can be fatal.

## Introduction

Methemoglobinemia is a rare but potentially fatal disorder of oxygen carrying capacity of hemoglobin [1]. Physiologically MetHb is present in less than 1% of the patients?. This level is maintained at a constant rate by two mechanisms. One is cytochrome *b5* reductase pathway and the other is nicotinamide adenine dinucleotide phosphate (NADPH) dependent MetHb reductase, requiring a cofactor methylene blue or riboflavin for activation [2]. Clinical presentation is directly related to the levels of methemoglobin, Table 1 [3]. The purpose of presenting this case is to emphasize the importance of considering rare causes of cyanosis in the presence of normal cardio-respiratory systems.

**Table 1:** Clinical symptoms correlation with MetHb levels

MetHb level (%)	Clinical Presentation
0–3	None
3–10	Cyanosis, asymptomatic
10–20	
20–50	CNS changes (headache,dizziness,confusion
50–70	MetabolicAcidosis, dysrhythmias.
➢ 70	fatal

## Case report

A 22-year-old young Asian lady presented to emergency dept. with short history of bluish discoloration of her skin, primarily over lips, tongue, hands and feet. She also had complaints of gradually progressive dyspnea, palpitation and apprehension. She denied any history of fever, cough or sputum production. For these complaints she visited different health facilities & was given nebulization, supplemental oxygen, but symptoms didn’t improve.

Past history included painful red nodules over extensor surface of lower limbs, which resolved with steroids use.. However they reappeared many times later and did not regress much with steroids afterwards. Later on she was diagnosed to have erythema nodosum, and was prescribed steroids once again.

On examination, she was dyspneic, centrally cyanosed with 86% O_2_saturation at room air. Rest of the examination was unremarkable. She was given supplemental oxygen via face mask but saturation didn’t improve more than 88%. All her laboratory investigations were normal Table 2 . Her ABGs at maximum O_2_ revealed PaO_2_ 88 (60–90 mmHg), pH 7.41 (7.36–7.46), PCO_2_ 38.2 (34-46 mmHg), HCO_3_ 26 (22-27 mEq/L). ECG as well as Cardiac enzymes were normal. CTPA done to rule out Pulmonary Embolism was also normal. No circulatory or ventilatory abnormality was found to explain cyanosis. Her cyanosis with normal PaO_2_ was the pointer to the diagnosis.

**Table 2:** Detailed Serial Blood CP Report

Indices	At Admission	3rdDay	7Th Day
Hb mg/dl	11.1	11.9	10.9
MCV fL	75.2	76.0	76.4
MCH mg/dl	26.7	25.8	26.1
TLC 10^3^/L	9.1	13.25	9.2
Platelets 10^3^/L	380	377	376

All her medical record along with history revealed, she has been taking Dapsone for treatment of her skin problem. This led to the final diagnosis of Dapsone induced Methemoglobinemia. Unfortunately MetHb level estimation is not possible in the country. She was treated with methylene blue and showed complete recovery. Methlene Blue was administered intravenously at dose of 1 mg/kg body weight. This dose was repeated once more and patient was later on discharged. She was doing fine on follow-up.

## Discussion

Oxygen in blood is carried by hemoglobin. When this hemoglobin gets oxidized (due to hereditary defects or by oxidative stress) it forms Methemoglobin, resulting in tissue hypoxia.

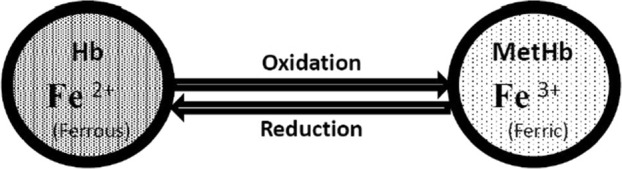



Our body has the capability to reduce this MetHb to Hb via certain enzymes such as cytochrome *b5* reductase. In its acquired form, exposure to various oxidizing agents can also result in conversion of normal Hb to MetHb. These oxidizing agents include drugs like benzocaine, lidocaine, prilocain, antibiotics like dapsone, choloroquine, nitrites, some toxins, aniline dyes etc. [2, 4]

In the case presented here Dapsone was found to be the cause of Methemoglobinemia. Dapsone is used as an antibacterial and anti-inflammatory agent. Dapsone also known as DDS (…) is more commonly used in dermatology. It is available as oral and topical formulations. Recently long acting injectable form of Dapsone has also gained importance. When orally administered, it is absorbed slowly and reaches its peak plama concentration in 4 h with a half like of 1.1 h. It is eliminated after approximately 30 h. The parenteral administration is given as monthly injection and under supervison as recommended by WHO [5].

Clinical presentation may vary from being asymptomatic to confusion, cardiovascular collapse and finally death [6]. Cyanosis usually appears when MetHb levels reach 10% or above [7]. Symptoms usually depend on MetHb level, however it may not correlate every time, therefore, for the diagnosis of this condition, high index of clinical suspicion is required [6]. Any patient whose O_2_ saturation does not improve with high flow supplemental O_2_ and an O_2_ saturation gap greater than 5% should raise the suspicion of MetHb [2].

The oxygen saturation gap is measured as percentage. It is the difference between oxyhemoglobin concentration recorded by a pulse oximeter from arterial oxygen concentration determined by a standard blood gas analyzer. Levels more than 5% are considered abnormal and calls for evaluationof Methemoglobinemia, sulfhemoglobinemia and carboxyhemoglobinemia [8].

Saturation gap in studied patient was 96.7%. Certain bed side tests like color of blood and ABGs help in diagnosing this condition. Color of blood is brown in patients with MetHb. Confirmation is done by direct estimation of methemoglobin levels and if required genetic analysis.

Methylene blue is so far the only specific antidote for treatment of symptomatic MetHb. Patients not responding to methylene blue might require blood or exchange transfusions. Ascorbic acid is also given to reduce its levels.

Limitation in this case was the definitive confirmation by estimation of MetHb levels,which is unfortunately not available in Pakistan. However, the fact that a high index of suspicion in face of certain clues is key to diagnosis, and if left untreated or not treated promptly, the condition can be life threatening. Also the fact that serum levels usually do not correlate exactly with the symptoms, treatment was given empirically in this case [2, 9].

## Conclusion

In conclusion, acquired MetHb is a rare blood disorder which can be fatal condition if left untreated. It should be considered in the differential diagnosis of central cyanosis in presence of normal ventilatory and circulatory systems.

## Data Availability

Please contact author for data request.
